# Age-associated network controllability changes in first episode drug-naïve schizophrenia

**DOI:** 10.1186/s12888-021-03674-5

**Published:** 2022-01-10

**Authors:** Biqiu Tang, Wenjing Zhang, Shikuang Deng, Jiang Liu, Na Hu, Qiyong Gong, Shi Gu, Su Lui

**Affiliations:** 1 Huaxi MR Research Center (HMRRC), Functional and Molecular Imaging Key Laboratory of Sichuan Province, Department of Radiology, West China Hospital of Sichuan University, No. 37 Guoxue Xiang, 610041 Chengdu, Sichuan China; 2 Research Unit of Psychoradiology, Chinese Academy of Medical Sciences, 610041 Chengdu, Sichuan China; 3 School of Computer Science and Engineering, University of Electronic Science and Technology of China, No. 2006 Xiyuan Avenue, West Hi-Tech Zone, 611731 Chengdu, China

**Keywords:** Schizophrenia, Network controllability, Functional dynamics, Structural connectivity, Age-related change, Psychoradiology

## Abstract

**Background:**

Recent neuroimaging studies revealed dysregulated neurodevelopmental, or/and neurodegenerative trajectories of both structural and functional connections in schizophrenia. However, how the alterations in the brain’s structural connectivity lead to dynamic function changes in schizophrenia with age remains poorly understood.

**Methods:**

Combining structural magnetic resonance imaging and a network control theory approach, the white matter network controllability metric (average controllability) was mapped from age 16 to 60 years in 175 drug-naïve schizophrenia patients and 155 matched healthy controls.

**Results:**

Compared with controls, the schizophrenia patients demonstrated the lack of age-related decrease on average controllability of default mode network (DMN), as well as the right precuneus (a hub region of DMN), suggesting abnormal maturational development process in schizophrenia. Interestingly, the schizophrenia patients demonstrated an accelerated age-related decline of average controllability in the subcortical network, supporting the neurodegenerative model. In addition, compared with controls, the lack of age-related increase on average controllability of the left inferior parietal gyrus in schizophrenia patients also suggested a different pathway of brain development.

**Conclusions:**

By applying the control theory approach, the present study revealed age-related changes in the ability of white matter pathways to control functional activity states in schizophrenia. The findings supported both the developmental and degenerative hypotheses of schizophrenia, and suggested a particularly high vulnerability of the DMN and subcortical network possibly reflecting an illness-related early marker for the disorder.

**Supplementary Information:**

The online version contains supplementary material available at 10.1186/s12888-021-03674-5.

## Background

Schizophrenia is increasingly conceptualized as a disorder with abnormal neurodevelopmental, or/and neurodegenerative connectivity trajectories [1, 2]. The neurodevelopmental hypothesis indicated that genetic and early-life environmental risk factors may alter neurodevelopmental synaptic plasticity (such as synaptogenesis and synaptic pruning) [3], and result in the concomitant disruption of balanced interplay between integration and segregation of both structural and functional brain networks [4], and ultimately alter neurodevelopment trajectories to the onset of schizophrenia during late adolescence or early adulthood [1, 5]. Supporting the neurodevelopment models, the neuroimaging studies have revealed aberrant developmental trajectories in schizophrenia, for example, the delayed development of brain connectivity [6] and dysregulated maturation of the functional connectivity [7].

On the other hand, the neurodegenerative models identify schizophrenia as a progressive neurodegenerative disease [2, 8]. The prior findings of accelerated aging in gray matter [9, 10], white matter [11, 12] and large-scale functional brain networks in schizophrenia [13] provided evidence supporting the neurodegenerative model underlying schizophrenia. In this context, schizophrenia might be a disorder that incorporates both neurodevelopmental and neurodegenerative processes, but which brain regions/networks are specific to each of them remains to be established.

Notably, the alterations of the brain’s structure (such as myelination and white matter integrity) have critical implications for function which reflects the coordinated neural activity between different brain regions [14, 15]. Understanding the mechanism by which structure evolves to support the coordination of neural activity, would have far-reaching implications for our understanding of vulnerabilities to schizophrenia.

The recently developmental network control theory can simulate consequences of structural connections on function dynamics [16]. In the network control model, the brain is constructed of networks defined by structural connection, while the neural state is defined by a linear dynamic model [16, 17]. Network controllability is a structural predictor of the capability of steering network states to any configuration through external control [16, 18]. Average controllability as a network controllability metric quantifies capacity of brain regions or networks to steer the system to many easily reachable states, is a structural phenotype predicted to facilitate small changes in brain state [16]. The network control theory has been used in uncovering the underlying structural mechanism of macroscale brain dysfunction in multiple psychiatric and neurological disorders, such as bipolar disorder [19], schizophrenia [20], Parkinson’s disease [21] and epilepsy [22]. A recent study on network controllability of psychosis spectrum disorders further indicated that average controllability can predict positive psychosis spectrum symptoms better than all other network connectivity characteristics, considering the impact of both direct and indirect structural connectivity on the spread of activity [23]. In healthy controls, it has also been shown that network controllability measurements dynamically change during neurodevelopment [18], and support the development of executive function during youth [24]. However, to our knowledge, no studies have investigated age-related changes of network controllability in untreated schizophrenia patients to date.

The present work aimed to characterize age-related network controllability alterations in a relatively large sample of drug-naïve schizophrenia patients. As network controllability abnormality has been suggested as illness-trait of brain functioning in psychiatric disorder, characterizing the changes along aging might be more informative for locating the changes specific to neurodevelopmental/neurodegenerative processes. In the exploratory analysis, average controllability alterations were related to clinical features of schizophrenia. We hypothesized that schizophrenia patients would show differences in age-related trajectories of network control properties relative to healthy controls.

## Methods

### Participants

This present study included 175 patients with drug-naïve schizophrenia (SCZ) and 155 healthy controls (HCs) between 16 and 60 years of age. All participants were right-handed and of Han ancestry, and recruited at the West China Hospital from September 2005 to May 2014 (see Table 1 for demographic and clinical characteristics of all participants). Diagnosis of schizophrenia was determined using the Structured Clinical Interview for DSM-IV Axis I Disorders (SCID). Illness onset was determined using the Nottingham Onset Schedule [25], while psychiatric illness severity was assessed using the Positive and Negative Syndrome Scale (PANSS) and the Global Assessment Function (GAF) scale. Healthy controls were recruited from local communities through advertisement, and had no personal history or known history of major psychiatric illness in first-degree relatives. Other exclusion criteria for both two groups included current or past substance abuse, current pregnancy, significant systemic medical illness that might impact brain measures, history of neurological disorder or neurosurgery and MRI contraindications.

**Table 1:** Demographic and clinical information of drug-naïve schizophrenia patients and healthy control participants

	Schizophrenia (N = 175)	Healthy controls (N = 155)	Statistics	
	Mean ± SD	Mean ± SD	t or χ^2^	p value
Age (Years)	28.68 ± 10.86	27.02 ± 11.39	1.36	0.176
Sex (M/F)	81/94	71/84	0.01	0.931
Education years	11.61 ± 3.54	12.41 ± 3.44	2.09	0.037
DUP (Months)	33.92 ± 74.56	NA	NA	NA
GAF scores	30.19 ± 10.87	NA	NA	NA
PANSS scores				
Total	89.95 ± 16.20	NA	NA	NA
Positive	24.63 ± 6.31	NA	NA	NA
Negative	19.22 ± 8.01	NA	NA	NA
General psychopathology	46.11 ± 9.18	NA	NA	NA

***Abbreviations:*** *SD *standard deviation, *M *male, *F *female, *DUP *duration of untreated psychosis, *GAF *global assessment of functioning scale, *PANSS *positive and negative syndrome scale, *NA *not applicable

Notably, drug-naive schizophrenia patients were identified by a regional Mental Health Screening Program designed to identify and then provide psychiatric care to individuals with serious but untreated mental illness. There are 17 drug-naïve patients with long-term schizophrenia aged from 36 to 60 years. Most lived in small rural villages, and had not received any prior psychiatric treatment for various reasons, primarily because of parental concern about family stigma; a lack of understanding of the severity of the mental illness; poor socioeconomic conditions that limited travel and funds for medical care; and conflicts with physicians when the patient was first brought to medical attention close the time of illness onset. Each patient had been cared for and sheltered in their parents’ home without medical care through the course of their illness.

### MRI data acquisition

All MRI scans including diffusion tensor imaging (DTI) and high-resolution T1-weighted imaging were conducted on a 3T MRI scanner (EXCITE, General Electric). The DTI data were acquired using a single-shot spin-echo echo-planar image sequence: repetition time (TR) = 1000 ms, echo time (TE) = 70.8 ms, field of view (FOV) = 240 mm×240 mm, voxel size = 1.8 × 1.8 × 3.0 mm^3^, and slice thickness = 3.0 mm with no interslice gap. Each DTI data set included 15 non-collinear directions (b = 1000 s/mm^2^) and a reference image without diffusion weighting (b = 0 s/mm^2^). The high-resolution T1-weighted images were obtained using a spoiled gradient recall sequence: TR = 8.5 ms, TE = 3.4 ms, flip angle = 12°, Inversion time = 400 ms, FOV = 240 mm×240 mm, voxel size = 0.47 × 0.47 × 1 mm^3^, slice thickness = 1 mm with no gap and 156 slices. Two experienced neuroradiologists (Tang and Zhang) separately inspected MR images to exclude data with gross brain abnormalities or visible artifacts.

### Data preprocessing and network construction

Preprocessing of DTI data was conducted with the Pipeline for Analyzing Brain Diffusion Images toolkit (PANDA, http://www.nitrc.org/projects/panda), including head motion correction, eddy current correction, the voxel-wise tensor matrix and diffusion tensor metrics (such as fractional anisotropy (FA), the primary measure of interest) calculation. We used the atlas of Lausanne 2008 [26, 27] to parcellate the entire brain into 220 cortical and 14 subcortical regions (the bilateral thalamus proper, caudate, putamen, pallidum, accumbens area, hippocampus, and amygdala).

The FA is the most widely used DTI measures, could primarily reflect the myelin integrity, and quantify the fiber integrity of the connections [28, 29]. Thus, FA values between the end-nodes were used as the connectivity weight between two nodes, then a 234 × 234 weighted matrix for each participant was obtained. To define priori network, each cortical Lausanne label was assigned to a functional system defined by Yeo et al. [30], by calculating the purity index for the 7-system parcellation and 234 brain regions from the Lausanne atlas. The purity index quantifies the maximum overlap of cortical Lausanne labels and 7-system parcellation [31]. The 7-system parcellation include Default Mode Network (DMN), Dorsal Attention Network (DA), Frontoparietal Control Network (FCN), Limbic Network (LN), Somatomotor Network (SMN), Visual Network (VN), Ventral Attention Network (VA). Subcortical nodes were assigned to an eighth network based on the prior study [31].

### Network controllability

The network control theory can simulate consequences of a network’s underlying structural topology on its function dynamics [15, 32, 33]. The present study applied the network control theory framework to calculate network controllability, following prior work [16, 18, 24, 25, 34–36], implemented in MATLAB with in-house packages and code. The two essential components of this approach are structural connections and a linear dynamic model which defines the activity state of the brain and describes dynamic transition between functional states [16, 17]. Base on the previous studies, we define structural connectivity networks by subdividing the entire brain into 234 anatomically distinct brain areas (network nodes) on the basis of tractography data in humans [18, 26]. We define the of neural dynamics by drawing on prior models predicting human resting state functional dynamics from structural connectivity [15, 16]. The validity of this model offering mechanistic predictors of network dynamics on the basis of diffusion data rather than real fMRI data has previously been demonstrated based on extensive prior work in human systems neuroscience [15]. The details of dynamic model are provided in Supplementary Materials.

Network controllability refers to the possibility of steering network states to any configuration through external control. Average controllability as a network controllability metric quantifies capacity of brain regions or networks to steer the system to many easily reachable states [16]. Regions or networks with higher average controllability are thought to have greater potential impact on network dynamic control [16]. Detailed derivation of average controllability is provided in Supplementary Materials.

### Statistical analyses

#### Age-related changes of network controllability

Statistical analyses were performed using the SPSS software (Statistical Product and Service Solutions for Windows version 24.0, https://www.ibm.com/analytics/spss-statistics-software). In the primary analysis, linear model was adopted to examine the age-related changes of network controllability based on the prior work showing white matter (WM) microstructure changed linearly with age in healthy controls [37]. During the analysis, general linear model (GLM) was used to predict WM network controllability with diagnosis (SCZ, HC), age, and diagnosis by age interaction being the dependent variables, while sex and education years were treated as covariates. The GLM was repeated for each one of the 8 networks and 234 nodes respectively, the findings were corrected by false discovery rate (FDR) to preserve a p < 0.05 experiment-wise threshold. For the networks or nodes with significant diagnosis by age interactions on network controllability, which indicates that there are significant differences in the modeled age-related changing trajectories between diagnostic groups, regression curves were established to precisely depict the associations between network controllability and age in the patient group and control group respectively, with sex and education years as covariates.

Subsequently, to further map the age-related patterns of network controllability in the SCZ, we also used the stage-specific approach that has been used in previous research [7]. Previous lifespan studies showed that peak FA values and minimum mean diffusivity (MD) values mostly occur before 35 years [38]. Therefore, in the present study, 35 years was chosen as a cut-off to divide all participants into two age subgroups (adolescents and young adults: age 16-35 years, 133 patients, 116 controls; late adults: age 36-60 years, 42 patients, 39 controls, see Table S1 for demographic and clinical characteristics of participants in each age subgroup). Then, we assessed the interaction effect between diagnosis and age subgroup (16-35 years, 36-60 years) on network controllability, as well as the interaction effect between diagnosis and age on network controllability in each age subgroup using GLM. Sex and education years were controlled in these analyses. For explanatory purposes, we performed the subgroup analysis in networks and regions with significant age-related controllability alterations which found in the primary analysis, thus the findings of subgroup analysis were not corrected for multiple comparisons for these heuristic analyses.

#### Correlations between network controllability and clinical characteristics

For networks and nodes with significant age-related network controllability changes, two-tailed partial correlations were performed to examine the associations between the network controllability with duration of untreated psychosis (DUP) and symptom severity, controlling age, sex and education years. Statistical significance of correlations was set at p < 0.05 after correction with FDR.

## Results

### Age-related changes of network controllability

**Fig. 1:**
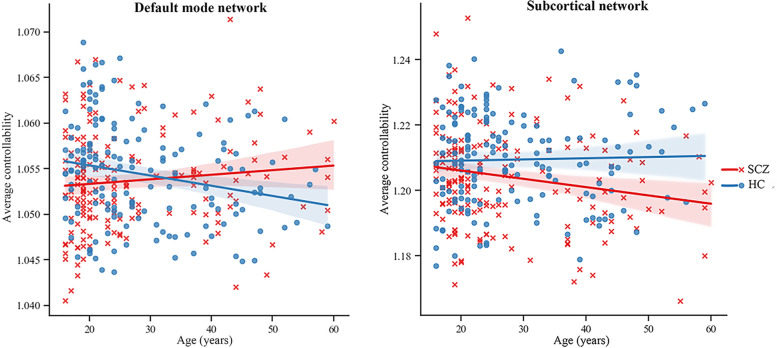
Linear modeling of age effects on network-level average controllability in drug-naïve schizophrenia patients and healthy controls. Diagnosis by age interactions were significant for the default mode network (p = 0.040) and the subcortical network (p = 0.030, uncorrected). Abbreviations: SCZ-schizophrenia, HC-healthy control

At the network level, there was a significant main effect of diagnosis by age interaction (F = 7.94, p = 0.040) and diagnosis (F = 9.30, p = 0.016, SCZ < HC) on the average controllability of DMN. Further analysis revealed that the patients with schizophrenia had lower average controllability than healthy controls, but showed no significant age-related change on average controllability of DMN network (r = 0.11, p = 0.148), while healthy controls showed significant age-related decline on average controllability of DMN (r = −0.17, p = 0.036) (Fig. 1). We also observed significant diagnosis by age interaction (F = 4.76, p = 0.030, uncorrected) on the average controllability of subcortical network. Further analysis revealed that the patients with schizophrenia showed significant age-related decline on average controllability of subcortical network (r = −0.19, p = 0.011), while healthy controls showed no significant age-related change (r = 0.11, p = 0.195) (Fig. 1).

**Fig. 2:**
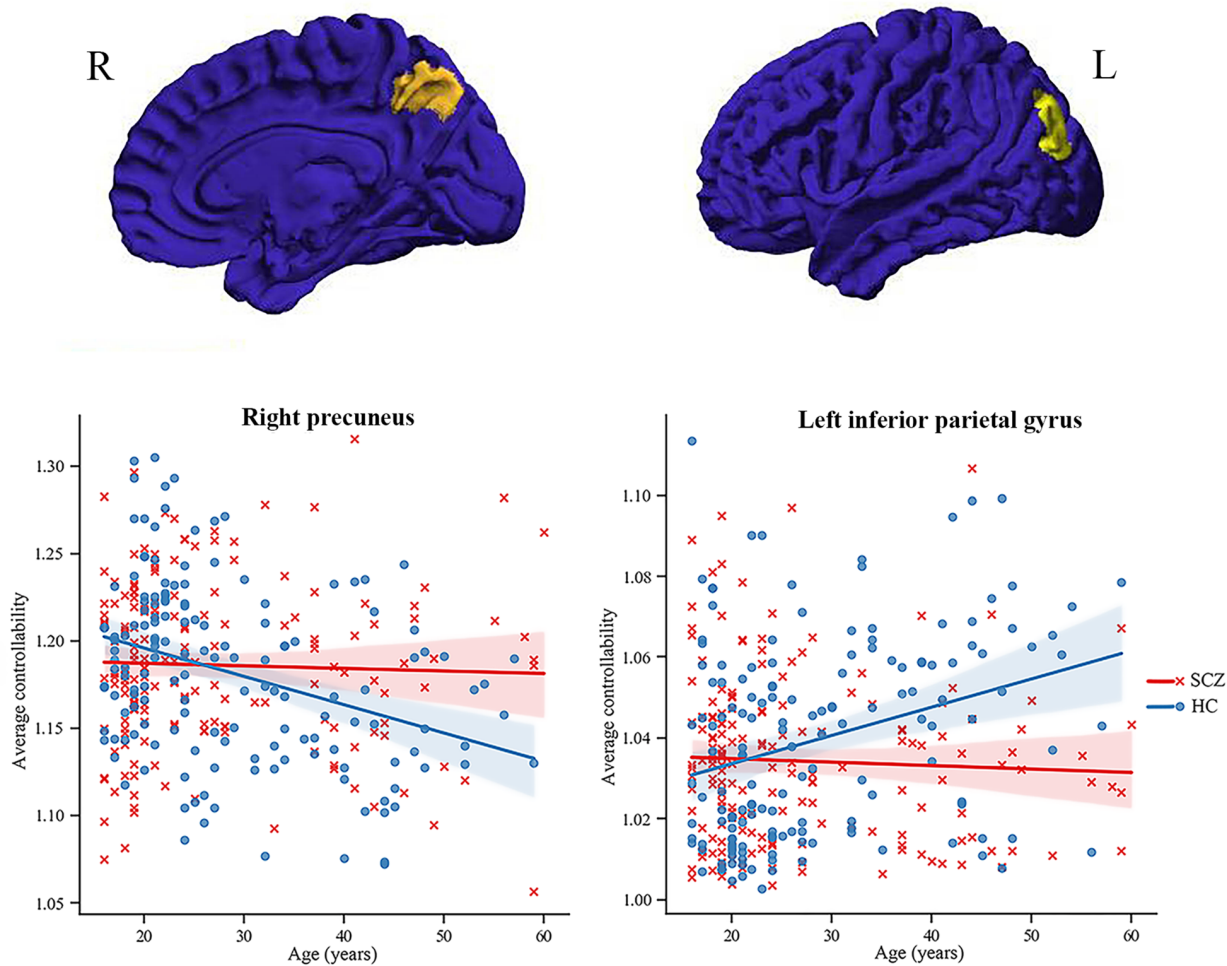
Linear modeling of age effects on node-level average controllability in drug-naïve schizophrenia patients and healthy controls. Diagnosis by age interactions reached significance for the left inferior parietal gyrus (p < 0.001) and the right precuneus (p = 0.002, uncorrected) at the level of node. Abbreviations: SCZ-schizophrenia, HC-healthy control, R-right, L-left

At the nodal level, our findings revealed a significant main effect of diagnosis by age interaction (F = 13.80, p < 0.001) and diagnosis (F= 13.78, p < 0.001, SCZ < HC) on the average controllability of left inferior parietal gyrus. Further analysis indicated a higher average controllability of this region in healthy controls than schizophrenia patients, accompanied by a significant age-related increase of the network controllability measure in HC (r = 0.21, p = 0.009) and no significant age-related change in SCZ (r = -0.06, p = 0.448) (Fig. 2). In addition, there was significant interaction of diagnosis by age (F = 9.44, p = 0.002, uncorrected) on the average controllability of the right precuneus, driven by significant age-related decrease on average controllability of the right precuneus in HC (r = −0.28, p = 0.001) and no significant age-related change in SCZ (r = 0.08, p = 0.269) (Fig. 2).

Since the lower education years in schizophrenia patients might be directly related to the illness, we also replicated the age-related controllability analyzes in schizophrenia without controlling education years. Notably, the main findings remained stable with or without controlling education years (see Supplementary Materials for details).

In line with our findings of diagnosis by age interaction effects in the primary analysis, the findings of subgroup analysis indicated that the subcortical network, DMN and the left inferior parietal gyrus were the main network/regions with age-related average controllability changes in schizophrenia (see Supplementary Materials for details, Fig. S1).

### Associations between network controllability and clinical characteristics

In the patient group, there were no significant correlation between average controllability measures and DUP, GAF scores, or PANSS scores after controlling age, sex and education years.

## Discussion

The network control theory can offer a mechanistic explanation for how the brain moves between functional states on the basis of white matter microstructure [16]. We use average controllability, as structural predictor of brain dynamics, to quantify ability of specific brain regions or networks to steer the system to many easily reachable states. The present study combined diffusion MRI and a network control theory approach to investigate the aberrant age-related alterations of average controllability in drug-naïve schizophrenia patients. Compared with controls, schizophrenia patients showed differences in age-related model of average controllability in DMN and subcortical network at the level of network, as well as the right precuneus and left inferior parietal gyrus at the nodal level. Notably, the schizophrenia patients demonstrated the lack of age-related decrease on average controllability of DMN network, as well as the right precuneus (a hub region of the DMN), suggesting an aberrant narrowing or specialization of their preferred control roles with age, providing additional support for the neurodevelopmental hypothesis of schizophrenia. Furthermore, the schizophrenia patients demonstrated accelerated age-related decline of average controllability in the subcortical network, indicating accelerated age-related decline of ability of the subcortical network to shift brain function into the optimal context-appropriate brain state, and supporting the neurodegenerative model. Taken together, the findings suggest that the DMN and subcortical network may be particularly vulnerable to the maturational and degenerative process in schizophrenia respectively, and contribute to the growing evidence that network controllability metrics might represent illness-related early biomarkers.

### Age-related changes of network controllability

Healthy controls in the present study displayed an age-related decline in average controllability of the DMN, as well as the right precuneus, which may be the result of a fine-tuning of the structural organization in an optimized way for diverse functional dynamics with maturation [18]. In contrast, schizophrenia patients didn’t display age-related change in the average controllability metrics of DMN, possibly mirroring the dysregulated maturational processes, in which underlying anatomical connections changes caused by aberrant synaptic pruning and myelination lead to altered functional integration and segmentation of DMN [14, 15]. Previous studies revealing functional disturbance of the DMN in patients with schizophrenia and the relatives, suggested a genetic factor for the impairments of functional development on DMN [39, 40]. The abnormal network controllability changes of DMN revealed the disruption of the functional balance between the DMN and other task-relevant brain systems which needed to bring cognitive resources to bear to respond adaptively to current environmental demands [41–43].

Consistent with previous findings, the present study showed that network controllability metric of the subcortical network in healthy controls did not significantly change with age, indicating that subcortical regions are stable controllers [18]. Relative to controls, the age-related decrease on average controllability of subcortical network in schizophrenia patients may indicate dysregulated degenerative trajectories in schizophrenia, consistent with accelerated aging of subcortical structures in schizophrenia [9, 44]. The dysregulated degenerative trajectory of WM network controllability in schizophrenia may result from the underlying anatomical changes and subcortical dopamine dysregulation via aberrant reactions within the stress-response circuity [5, 45].

Compared with controls, the lack of age-related increase on average controllability of the left inferior parietal gyrus in schizophrenia patients may suggest a different pathway of brain development consistent with similar findings of deficits in the parietal cortices in adolescent siblings of patients with schizophrenia [46].

### Associations between network controllability and clinical characteristics

Importantly, aberrant network controllability metrics were not associated with DUP, suggesting that alterations in these networks and regions might reflect early markers of schizophrenia. The present study did not observe associations between brain network controllability properties and symptom severity, possibly reflecting that network controllability might not directly relate to the current symptom at the onset of the disorder and might be relevant to the illness trait of schizophrenia.

### Limitations

There are several limitations that should be considered when interpreting the current results. First, our data is cross-sectional, limiting the ability to make causal inferences about network controllability changes across the lifespan of schizophrenia patients. The longitudinal experimental designs can trace the precise trajectories of brain changes theoretically throughout the course of illness. However, such studies will inevitably incorporate the confounding effects of antipsychotic drugs. Alternatively, cross-sectional designs with a large number of participants can also model age-related brain changes, especially preferable when treatment effects need to be excluded, and have frequently been used to investigate progressive brain changes [9, 11]. Second, schizophrenia typically appears in adolescents and young adults, and our samples were not perfectly distributed across the entire lifespan--the number of young adults was greater than thatof older participants. The uneven distribution of age in our sample may affect the mapping of age-related network controllability trajectories. Future studies with larger samples, especially with more older participants, are required to validate our findings. Third, there is no consensus of approach for calculating network metrics, and we used the FA values as the structural network connectivity weight. Other measures such as the MD or number of fibers could also be considered as weighting factors. Finally, some other clinical features such as cognition and blood tests were not systematically assessed, future work is required to comprehensively determine the clinical correlates and mechanisms of our findings.

## Conclusions

By modeling network controllability with age in untreated schizophrenia, the present study illustrated aberrant age-related changes in ability of white matter pathways to control functional activity states relative to healthy controls. The findings supported both the developmental and degenerative hypotheses of illness course of schizophrenia, and suggested a particularly high vulnerability of the DMN and subcortical network possibly reflecting an illness-related early marker for the disorder.

## Supplementary Information

**Additional file 1.**

## Data Availability

The datasets generated and/or analyzed during the current study are not publicly available due to that they contain patients’ personal information and our hospital has strict data sharing policy, but they are available from the corresponding author on reasonable request.

## Abbreviations

DMN: default mode network
SCZ: schizophrenia
HC: healthy controls
PANSS: Positive and Negative Syndrome Scale
GAF: Global Assessment Function scale
DTI: diffusion tensor imaging
TR: repetition time
TE: echo time
FOV: field of view
FA: fractional anisotropy
WM: white matter
GLM: general linear model
FDR: false discovery rate
MD: mean diffusivity
DUP: duration of untreated psychosis
