# Supplementary material for: Age-associated network controllability changes in first episode drug-naïve schizophrenia
Source: BMC Psychiatry. 2022 Jan 10;22:26. doi: 10.1186/s12888-021-03674-5 (PMC8744281; doi:10.1186/s12888-021-03674-5)
Supplement: Supplementary file 1 — Additional file 1. [file 12888_2021_3674_MOESM1_ESM.docx]

# Supplementary Materials

**Age-associated network controllability changes in first episode drug-naïve schizophrenia**

# Supplementary Methods

## *Dynamical model*

Based on the previous studies [1], neural states can be mathematically described as simulated states (x) of network with k nodes overtime steps t using a simplified noise-free linear discrete-time and time-invariant model:

$$x\left( t+1 \right)=Ax\left( t \right)+B_{K}u_{K}\left( t \right),$$

where $x\left( t \right)$ is a vector that describes the state of all brain regions at time $t$, while matrix *A* is a structural connection matrix. *K* is the set of nodes that can be controlled independently. The input matrix defines the control nodes. The $u_{K}\left( t \right)$ is the control signal injected into the network via the control nodes, while *B* is the input matrix.

## *Average Controllability*

Subsequently, the influence of each region on brain function is quantified using the metric of controllability. Average controllability quantifies capacity of brain regions or networks to steer the system to many easily reachable states [1]. It is calculated as the average energy (or effort) required to reach all possible states of the system.

As in previous work [1, 2], we used $Trace(W)$ as the measure of average controllability, where,

$$W=\sum_{\tau=0}^{T-1} A^{\tau}BB^{⊺}\left( A^{⊺} \right)^{\tau}.$$

Here, *B* is set equal to one canonical vectorto calculate the corresponding *W*, and this process is repeated for all nodes [1]. $⊺$ denotes the transpose operation, $\tau$ indicates the time step of the trajectory, and $T$ denotes the time horizon, which is set to infinity. Average controllability is computed for each node in $A$ separately.

# Supplementary Results

## *Age-related changes of network controllability without controlling education years*

At the network level, there was a significant main effect of diagnosis by age interaction (F = 7.91, p = 0.040) and diagnosis (F = 9.47, p = 0.016, SCZ < HC) on the average controllability of DMN. We also observed significant diagnosis by age interaction (F = 4.81, p = 0.029, uncorrected) on the average controllability of subcortical network. At the nodal level, our findings revealed a significant main effect of diagnosis by age interaction (F = 13.37, p < 0.001) on the average controllability of left inferior parietal gyrus. In addition, there was significant main effect of age (F = 12.95, p < 0.001) and interaction of diagnosis by age (F = 8.94, p = 0.003, uncorrected) on the average controllability of the right precuneus.

## *Age-related changes of network controllability in subgroup analysis*

The p values of subgroup analysis reported below were uncorrected. There was a significant interaction effect between diagnosis and age subgroup on average controllability in the subcortical network (F = 4.63, p = 0.032). Post hoc analysis demonstrated the average controllability of subcortical network was lower in the older than young SCZ (p = 0.010), and demonstrated the average controllability of subcortical network was lower in the older SCZ than older HC (p = 0.001). There also was a significant interaction effect between diagnosis and age subgroup on average controllability in the visual network (F = 5.80, p = 0.017). The post-hoc analysis demonstrated the average controllability of visual network was higher in the young SCZ than the young HC group (p = 0.001). We observed a significant diagnosis by age interaction (F = 3.53, p = 0.061) between diagnosis and age subgroup on average controllability in the DMN. In addition, post hoc analysis demonstrated the average controllability of DMN was slightly lower in the young SCZ than the young HC group (p = 0.053). At the level of node, there was a significant interaction between diagnosis and age subgroup on average controllability in the right precuneus and the left inferiorparietal gyrus (F = 4.61, 12.60, respectively, all p < 0.05). The post-hoc analysis demonstrated the average controllability of these two regions was higher in the older HC than the young HC group (all p < 0.05), and lower in the older SCZ than the older HC group (all p < 0.05).

In addition, subgroup analysis showed a significant main effect of diagnosis by age interaction (F = 8.10, p = 0.005) on the average controllability of DMN in the young subgroup, but no significant main effect of diagnosis by age interaction (F = 0.02, p = 0.903) in the older subgroup. Further analysis revealed that the young patients with schizophrenia showed a significant age-related increase trend on average controllability of DMN network (r = 0.17, p = 0.051), while young healthy controls showed significant age-related decline trend on average controllability of DMN network (r = −0.17, p = 0.064) (Figure S1).

# References

1. Gu S, Pasqualetti F, Cieslak M, Telesford QK, Yu AB, Kahn AE, Medaglia JD, Vettel JM, Miller MB, Grafton ST et al: Controllability of structural brain networks. Nat Commun 2015, 6:8414.

2. Muldoon SF, Pasqualetti F, Gu S, Cieslak M, Grafton ST, Vettel JM, Bassett DS: Stimulation-based control of dynamic brain networks. PLoS Comput Biol 2016, 12(9):e1005076.

# Table

**Table S1**. Demographic and clinical information of drug-naïve schizophrenia patients and healthy control participants in each age subgroup.

| **Characteristic** | **Schizophrenia**  **(16-35 years old)** | **Healthy controls**  **(16-35 years old)** | Statistics | | **Schizophrenia**  **(36-60 years old)** | **Healthy controls**  **(36-60 years old)** | Statistics | |
| --- | --- | --- | --- | --- | --- | --- | --- | --- |
|  | **N = 133, Mean ± SD** | **N = 116, Mean ± SD** | t or χ^2^ | p value | **N = 42, Mean ± SD** | **N = 39, Mean ± SD** | t or χ^2^ | p value |
| Age (Years) | 21.33 ± 4.42 | 23.21 ± 5.16 | 3.09 | 0.002 | 45.02 ± 7.15 | 44.97 ± 5.81 | 0.03 | 0.973 |
| Sex (M/F) | 60/73 | 62/54 | 1.72 | 0.189 | 21/21 | 9/30 | 6.29 | 0.012 |
| Education years | 12.64 ± 2.64 | 13.67 ± 2.57 | 3.12 | 0.002 | 8.33 ± 4.02 | 8.65 ± 2.93 | 0.22 | 0.830 |
| DUP (Months) | 11.72 ± 21.47 | NA |  | NA | 109.13 ± 125.89 | NA |  | NA |
| GAF scores | 30.73 ± 11.08 | NA |  | NA | 26.50 ± 8.72 | NA |  | NA |
| PANSS scores |  |  |  |  |  |  |  |  |
| Total | 88.69 ± 16.98 | NA |  | NA | 94.35 ± 12.35 | NA |  | NA |
| Positive | 24.31 ± 6.56 | NA |  | NA | 25.76 ± 5.29 | NA |  | NA |
| Negative | 18.53 ± 8.08 | NA |  | NA | 21.62 ± 7.36 | NA |  | NA |
| General psychopathology | 45.86 ± 9.01 | NA |  | NA | 46.97 ± 9.85 | NA |  | NA |

Abbreviations: SD-standard deviation, M-male, F-female, DUP-duration of untreated psychosis, GAF-global assessment of functioning scale, PANSS-positive and negative syndrome scale, NA-not applicable.

# Figure

**Figure S1**. Linear modeling of age effects on average controllability in drug-naïve schizophrenia patients and healthy controls within each age subgroup.


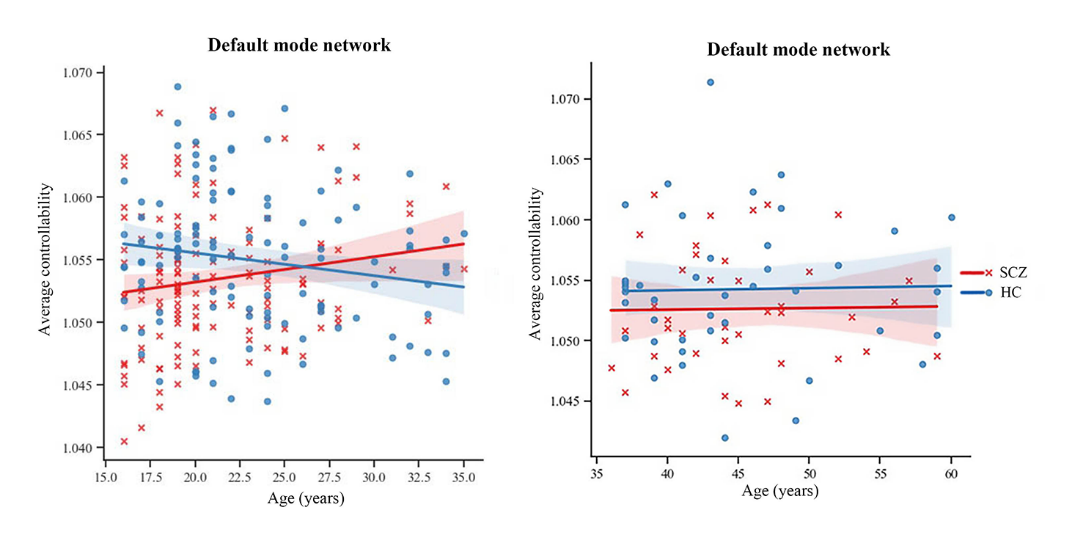


Subgroup analysis showed a significant main effect of diagnosis by age interaction (F = 8.10, p = 0.005) on the average controllability of default mode network (DMN) in the young subgroup, but no significant main effect of diagnosis by age interaction (F = 0.02, P = 0.903 in the older subgroup.

Abbreviations: SCZ-schizophrenia, HC-healthy control.
